# Neural risk factors that predict the future onset of binge eating or compensatory weight control behaviors: A prospective 4-year fMRI study

**DOI:** 10.1017/S0033291724003337

**Published:** 2025-02-17

**Authors:** Eric Stice, Sonja Yokum, Jeff Gau, Heather Shaw

**Affiliations:** 1 Department of Psychiatry and Behavioral Sciences, Stanford University; 2 Oregon Research Institute

**Keywords:** eating disorders, neural vulnerability, parental history, prospective, risk factors

## Abstract

**Background:**

We conducted a prospective study to advance knowledge of biological factors that predict the future onset of binge eating and compensatory weight control behaviors because few biological risk factors for eating pathology have been identified.

**Methods:**

Adolescent girls free of binge eating or compensatory behaviors (*N* = 88; *M*_age_ = 14.5; [SD = 0.9]) completed functional magnetic resonance imaging tasks assessing individual differences in neural responsivity hypothesized to increase risk for onset of binge eating and compensatory behaviors, along with additional self-report measures, and were assessed over a 4-year follow-up.

**Results:**

Elevated responsivity of regions implicated in attention and valuation (dorsal anterior cingulate cortex; ventromedial prefrontal cortex) to thin models and lower responsivity of a reward valuation region (caudate) to anticipated milkshake tastes (which correlated with feeling fat) predicted the future onset of binge eating or compensatory behaviors over 4-year follow-up. Parental history of binge eating and compensatory behaviors, emotionality, weight/shape overvaluation, feeling fat, and elevated BMI also predicted the future onset of binge eating or compensatory behaviors.

**Conclusions:**

The evidence that elevated attentional bias for, and valuation of the thin ideal, in combination with lower valuation of high-calorie foods, predicted the future onset of eating-disordered behaviors are novel findings. The evidence that weight/shape overvaluation, feeling fat, elevated body mass, emotionality, and parental history of eating pathology predicted the future onset of eating-disordered behaviors extend past findings.

Eating disorders affect 13% of women and are characterized by distress, impairment, suicide, and morbidity (Allen et al., 2013; Dakanalis et al., 2017; Stice et al., 2013b; Swanson et al., 2011). It is vital to identify risk factors that predict the future onset of eating disorder symptoms that crosscut eating disorders because this should advance etiologic theory, inform the content of prevention programs, and identify high-risk subgroups to target with selective prevention programs, which is critical because 80% of individuals with eating disorders do not receive treatment (Swanson et al., 2011).

Five prospective studies have examined baseline risk factors that predict the future onset of binge eating or compensatory weight control behaviors. Elevated overvaluation of weight/shape in determining self-worth, perceived pressure to be thin, pursuit of the thin appearance ideal, body dissatisfaction, body mass, dieting, negative affect, emotional eating, modeling of eating disorder symptoms, and deficits in peer social support predicted the future onset of binge eating (Chen et al., 2009; Sinclair-McBride & Cole, 2017; Stice & Agras, 1998; Stice et al., 2002). Elevated perceived pressure for thinness, body dissatisfaction, dieting, negative affect, and perfectionism predicted the future onset of compensatory weight control behaviors (Jones & Crowther, 2013; Stice & Agras, 1998). Similar risk factors and prodromal symptoms predicted the future onset of eating disorders (Allen et al., 2014; Dakanalis et al., 2017; Jacobi et al., 2011; Rohde et al., 2015; Stice et al., 2017; Stice et al., 2021a).

Although these studies have advanced knowledge of eating disorder risk factors, few prospective studies have identified biological variables that increase the risk for eating pathology. Neuroimaging may advance knowledge of risk processes for eating pathology. We, therefore, conducted an exploratory study in which we recruited 88 adolescent girls with and without parental history of recurrent binge eating or recurrent compensatory weight control behaviors, which was designed to capture parental history of threshold/subthreshold anorexia nervosa [AN], bulimia nervosa [BN], binge eating disorder [BED], and purging disorder [PD]), assessed several biological and other putative eating disorder risk factors at baseline, and conducted annual assessments over a 4-year follow-up. Children of parents with a history of eating disorders show a 3.7-fold increased incidence of future onset of eating disorders (Bould et al., 2015; Lydecker & Grilo, 2016). We initiated this hypothesis-generating study to provide data regarding biological and other types of risk factors that predict the future onset of binge eating or compensatory weight control behaviors over long-term follow-up.

This unique high-risk prospective risk factor design addresses two distinct research questions that are both useful for advancing knowledge of etiologic processes. First, it provides a way of detecting risk signatures at baseline among youth at high-risk versus low-risk for eating disorders before they show the emergence of eating pathology. Second, it provides a test of whether elevations in risk factors predict the future onset of eating pathology. If we can confirm that high-risk youth exhibit a particular risk signature (e.g., elevated reward region response to thin models) and also that this variable increases the risk for the future onset of eating pathology, it would provide compelling evidence for etiologic processes hypothesized to increase the risk for eating pathology.

We examined seven factors, in addition to parental history of eating pathology, that we hypothesized may increase the risk for the future onset of eating disorder behaviors. First, given that valuation of the thin ideal, overvaluation of weight/shape in determining self-worth, and fear of weight gain predicted the future onset of any eating disorder (Dakanalis et al., 2017; Rohde et al., 2015) and onset of AN, BN, BED, and PD (Stice et al., 2017; Stice et al., 2021a), we hypothesized that greater reward and attention region response to thin models would predict the future onset of binge eating and compensatory behaviors. Elevated responsivity in regions implicated in reward valuation and emotional salience (amygdala) and memory (parahippocampal gyrus) to thin models predicted the future persistence of eating disorder symptoms (Stice & Yokum, 2023). To our knowledge, no study has tested whether individual differences in neural response to thin models predict the future onset of binge eating or compensatory weight control behaviors. We also assessed self-reported valuation of the thin ideal, attractiveness ratings of thin models, overvaluation of weight/shape, fear of weight gain, feeling fat, and attentional bias for thin models to provide broader coverage of this risk construct. Parental-history-positive versus negative youth in the present sample reported greater overvaluation of weight/shape and feeling fat at baseline (Stice et al, 2021b). We could not locate any study that tested whether prodromal attitudinal eating disorder symptoms predicted the future onset of eating disorder behaviors.

Second, given that overeating and binge eating predicted the future onset of BN, BED, and PD (Stice et al., 2017; Stice et al., 2021a; Tanofsky-Kraff et al., 2011; Yamamiya & Stice, 2024) and that children who later developed BN evidenced greater overeating and overweight during childhood than children who did not (Micali et al., 2007), we hypothesized that elevated reward region response to tastes, anticipated tastes, and images of high-calorie foods would predict the future onset of binge eating or compensatory behaviors. Individuals with BN and BED showed greater responsivity of regions implicated in reward (medial OFC) to high-calorie food images and anticipated tastes of high-calorie foods than healthy controls (Schienle et al., 2009; Simon et al., 2016; Uher et al., 2004). Individuals recovered from BN showed greater striatum response to high-fat food taste and greater anterior insula, striatum, and amygdala response to high-sugar food taste than healthy controls (Ely et al., 2017; Oberndorfer et al., 2013). Parental-history-positive versus negative youth in the present sample showed elevated striatum response to anticipated tastes of chocolate milkshake at baseline (Stice et al, 2021b). No study has tested whether individual differences in neural response to tastes, anticipated tastes, or images of high-calorie foods predict the future onset of eating disorder symptoms. However, one study found that elevated ventral striatum to anticipated receipt of monetary reward predicted the future binge eating onset (Lowe & Bodell, 2024). We also assessed palatability ratings of high-calorie foods, liking and craving for high-calorie foods, attentional bias for high-calorie foods, and body mass.

Third, we hypothesized that weaker recruitment of inhibitory regions while attempting to inhibit a response to high-calorie foods and in response to tastes, anticipated tastes, and images of high-calorie foods would predict the future onset of binge eating or compensatory behaviors. Individuals with versus without BN or BED show inhibitory control deficits on food and nonfood go/no-go tasks, stop-signal tasks, and delay discounting tasks (Hege et al., 2015; Manwaring et al., 2011; Mobbs et al., 2011; Svaldi et al., 2014; Wu et al., 2013), less responsivity of inhibitory regions (dorso- and ventrolateral prefrontal cortex [dlPFC, vlPFC], ventromedial PFC [vmPFC], inferior frontal gyrus [IFG]) to high-calorie food images (Uher et al., 2004) and a food go/no-go task (Balodis et al., 2013; Hege et al., 2015), and impaired behavioral self-control and weaker recruitment of inhibitory regions (ACC, dlPFC, vlPFC, IFG) during a self-regulatory task (Marsh et al., 2011) and a nonfood go/no-go task (Skunde et al., 2016). We could not locate any studies that tested whether inhibitory control region responsivity predicted the future onset of binge eating or compensatory weight control behaviors. We also measured delay discounting for high-calorie foods to assess preference for immediate reward.

Fourth, because elevated negative affect and emotionality predicted the future onset of any eating disorder (Killen et al., 1996; Rohde et al., 2015) and AN, BN, BED, and PD (Stice et al., 2017; Stice et al., 2021a), we hypothesized that participants who showed greater limbic circuitry response to negative mood induction would be at increased risk for the future onset of binge eating or compensatory behaviors. Individuals with BN showed greater increases in negative affect and desire to binge during a negative mood induction than healthy controls (Tuschen-Caffier & Vögele, 1999). Further, parental-history-positive versus negative youth in the present sample reported greater emotionality at baseline (Stice et al, 2021b). However, we were unable to locate a study that tested whether elevated limbic region response to a negative mood induction predicted the future onset of binge eating or compensatory behaviors. We also assessed self-reported emotionality.

Fifth, we hypothesized that participants who exhibit greater caloric deprivation would be at risk for the future onset of binge eating or compensatory behaviors. Elevated self-reported dietary restraint predicted the future onset of any eating disorder (Rohde et al., 2015) and of BN, BED, and PD (Killen et al., 1996; Stice et al., 2017, 2021a) and weight suppression predicted the future onset of AN, BN, and PD (Stice et al., 2020). Yet, dietary restraint scores do not correlate with objective measures of caloric intake (e.g., Martin et al., 2005; Sysko et al., 2007), suggesting these scales are invalid. Fortunately, a greater number of hours since the last reported caloric intake correlated (mean *r* = .53) with greater fMRI-assessed reward region response to tastes, anticipated tastes, and images of high-calorie foods (Stice et al., 2013a), converging with experimental evidence that caloric deprivation increases reward region response to food images (Leidy et al., 2011). Results imply that repeatedly asking people how long it has been since last caloric intake via ecological momentary assessment (EMA) and averaging across responses may be a more valid measure of habitual caloric deprivation, potentially because it circumvents self-presentation biases. Parental-history-positive versus negative youth in the present sample reported greater habitual caloric deprivation based on EMA and lower liking of high-calorie foods, at baseline (Stice et al, 2021b). However, no study has tested whether participants who exhibit greater caloric restriction, based on EMA, are at increased risk for the future onset of binge eating or compensatory behaviors.

Sixth, birth complications correlated with a lifetime diagnosis of eating disorders (Cnattingius et al., 1999). Thus, we tested whether participants who experience more birth complications would show elevated the future onset of binge eating or compensatory behaviors.

## Methods

We recruited healthy female adolescents with or without a biological parental history of at least 6 binge eating episodes and/or at least 6 episodes of compensatory weight control behaviors (vomiting, laxatives/diuretic use, fasting, excessive exercise). We defined parental history of eating pathology in this fashion because we wanted to focus on eating-disordered behaviors versus cognitions because it seemed that parents would be better able to retrospectively report the frequency of these discrete behaviors and because these behaviors are germane to eating disorders characterized by binge eating (binge/purge AN, BN, BED) and unhealthy compensatory weight control behaviors (restricting AN, BN, PD). This definition approximates the frequency criteria for subthreshold BN, BED, or PD, which are associated with elevated psychosocial impairment, distress, service utilization, and obesity (Stice et al., 2013b). Although we did not require that these behaviors occur within a consecutive 3-month period, 62% reported at least 6 episodes of binge eating or compensatory behaviors during a 3-month period. During the 3-month period of peak symptoms, on average parents with a history of binge eating reported 23 binge episodes and parents with a history of compensatory behaviors reported 43 compensatory behaviors.

We sought to recruit 40 parental-history-positive and 40 parental-history-negative female adolescents because this would provide a power of .80 to detect at least medium between-group differences (Cohen, 1988). Mailings and fliers recruited biological females between 13 and 16 years of age for a study examining health and eating behaviors. We recruited 13- to 16-year-olds because this developmental window is immediately before the peak period of risk for onset of eating disorders (Stice et al., 2013b). Exclusion criteria were history of binge eating or compensatory weight control behaviors, fMRI contraindicators, use of psychotropic medications, illicit drugs, or weight loss drugs, serious medical problems, current Axis I diagnosis (including eating disorders; assessed with the Schedule for Affective Disorders and Schizophrenia for School Age Children – Epidemiologic Version 5 [K-SADS-E-5]; Orvaschel, 1994), and a BMI > 35 (due to scanner weight restrictions). Participants provided written assent and legal guardians provided written consent. At the first visit, participants completed a diagnostic interview, surveys, and computer tasks, and were familiarized with the fMRI paradigms. Participants returned for a second visit to complete fMRI scans. The Oregon Research Institute Institutional Review Board approved this study. Data were collected from 37 parental-history-positive youth and 51 parental-history-negative youth. Table 1 reports the demographics of the sample.

**Table 1.** Sample demographic characteristics (*N* = 88)

Age [Mean, (SD)]	14.5	(0.9)
Race and ethnicity (*n*, %)		
Asian	1	1.1
Hispanic	6	6.8
White	67	76.1
More than one race	14	15.9
Grade (*n*, *%*)		
7th	5	5.7
8th	17	19.5
9th	33	37.9
10th	25	28.7
11th	7	8.0
Maximum parental education (*n*, %)		
High school graduate	5	5.7
Some college	11	12.5
College graduate	45	51.1
Advanced degree	27	30.7
Onset of binge episode (*n*, % yes)	5	5.7
Onset of compensatory behaviors (*n*, % yes)		
Self-induced vomiting	5	5.7
Misuse of laxatives, diuretics, or other medications	0	0.0
Fasting (skipping at least two meals in a row)	15	17.0
Excessive exercise	1	1.1

Abbreviation: SD *= standard deviation.*

### Scan procedures and measures

Data were acquired using a Siemens Skyra 3 Tesla MRI scanner. The Supplementary Material section provides a detailed description of the fMRI data acquisition. Participants were asked not to eat or drink caffeinated beverages 3–4 hours before scans. Prior to the scan, they reported the last time they ate, hunger, and handedness. Participants showing the onset of binge eating or compensatory behaviors over follow-up versus no onset did not differ on scan time (onset: *M* = 14:22 ± 2:31; no onset: *M* = 14:20 ± 2.28, *t*(84) = .05, *p* = .96), hours since last caloric intake (onset: *M* = 3.21 ± 4.46; no onset: *M* = 2.80 ± 2.36, *t*(84) = .52, *p* = .61), hunger (onset: *M* = 8.38 ± 3.58; no onset: *M* = 8.83 ± 4.21, *t*(84) = −.41, *p* = .69), and handedness (onset = 82.4% right-handed; no onset = 91.5% right-handed, *t*(84) = 1.12, *p* = .27).

#### Model image paradigm

This paradigm assesses neural response to 20 thin models and 20 average-weight models (Stice et al., 2023). Participants were asked to think about the attractiveness of each model. Images were presented for 5 seconds (order randomized), followed by a 4–8 second jittered fixation cross. Images of models were selected to match on variables other than body weight (e.g., conventional attractiveness, race). Neural response to this paradigm predicted the future persistence of eating disorder symptoms (Stice & Yokum, 2023) and detected responses to eating disorder treatment (Stice et al., 2023).

#### Food image paradigm

This paradigm assesses neural response to 20 images of high-calorie binge foods and 20 images of glasses of water (Stice et al., 2010). Participants were asked to imagine eating the food and drinking the water. Images were presented for 5 seconds (order randomized), followed by a 4–8 second jitter with a fixation cross. Neural response to this paradigm predicted future weight gain (Stice et al., 2010) and detected responses to eating disorder treatment (Stice et al., 2023).

#### Food receipt paradigm

This paradigm assesses neural response to tastes and anticipated tastes of milkshake and tasteless solution (Stice et al., 2015). Stimuli were 2 cues (glasses of milkshake and water, 30 events each) that signaled impending delivery of either chocolate milkshake or tasteless solution (30 events each) during two runs (order randomized). Cues were presented for 2 s, followed by a blank jitter (1–7 seconds). Taste delivery (5 s) occurred 7–9 s after cue onset. Participants were instructed to swallow when cued (1 s). Neural response to this paradigm differentiated lean versus obese adolescents and predicted future weight gain (Stice et al., 2008; Stice et al., 2015). Prior to the scan, participants were asked to rate the pleasantness of the milkshake on a cross-modal visual analog scale (VAS) ranging from 0 (most unpleasant sensation ever) to 20 (most pleasant sensation ever). The mean milkshake pleasantness rating was 15.05 ± 2.79.

#### Food go/no-go paradigm

Participants completed an adapted version of our food go/no-go paradigm (Batterink et al., 2010). Participants were presented with images of desserts and vegetables during two runs (order randomized). In one run, participants were asked to press the button when they saw vegetables and inhibit the button press when they saw desserts. Instructions were reversed in the other condition. Each trial ended with a 2–4 second jittered fixation cross. There were 32 go-dessert trials, 31 go-vegetable trials, 13 no-go dessert trials, and 13 no-go vegetable trials. Neural and behavioral responses to this paradigm differentiated lean versus obese adolescents (Batterink et al., 2010).

#### Negative mood induction paradigm

We adapted the negative mood induction paradigm from Diers et al. (2014). Participants viewed 20 negative moods and 20 neutral mood self-referential statements accompanied by music (the Planets, Op. 32: VII. Neptune, the Mystic by Gustav Holst played at regular speed during neutral statements and Prokofiev’s Russia Under the Mongolian Yoke played at half speed during negative mood statements). Participants were asked to think about a time during which they felt like the statements, which were presented for 6 seconds. Following each statement, a fixation cross appeared (12 s), followed by a jitter (2–6 s). Before and after the task participants rated their mood. Mean mood (±*SD*) prior to the task was 70.0 ± 14.7 and 67.0 ± 17.7 after the task, a significant change: *t*(79) = 2.80, *r* = .30; *p* < .006 (Stice et al., 2021b). Neural response to the negative mood statements compared with the neutral mood statements elicited greater activation in regions implicated in negative mood processing (subgenual ACC; Ramirez-Mahaluf et al., 2018) at baseline (Stice et al., 2021b).

#### Adolescent eating pathology

The Eating Disorder Diagnostic Interview (EDDI) (Stice et al., 2017) assessed DSM-5 eating disorder symptoms over the past 12 months. We tested whether baseline overvaluation of weight/shape, fear of weight gain, and feeling fat predicted the future onset of binge eating or compensatory weight control behaviors. Inter-rater reliability was *κ* = .89 for overvaluation of weight/shape, *κ* = .86 for fear of weight gain, *κ* = .85 for feeling fat, *κ* = .94 for binge eating, and *κ* = .86 for compensatory behaviors (Stice et al., 2021a). The future onset of binge eating correlated *r* = .58 with future onset of compensatory weight control behaviors.

#### Parental eating pathology

Parental history of eating pathology was assessed with a version of the EDDI adapted to collect lifetime history at baseline. Biological parents were assessed to determine whether one or both had a history of 6 or more binge eating episodes and/or 6 or more compensatory behaviors. Adolescent girls with versus without parental history of eating pathology showed elevated reward region response to anticipated taste of chocolate milkshake, caloric deprivation, emotionality, overvaluation of weight/shape, and feeling fat at baseline (Stice et al., 2021b).

#### Pursuit of the thin ideal

The Ideal-Body Stereotype Scale–Revised assessed pursuit of the thin ideal (Stice et al., 2017). It has shown internal consistency (*α* = .91), 2-week test–retest reliability (*r* = .80), and predictive validity for BN, BED, and PD onset (Stice et al., 2017) (*α* = .88 at baseline).

#### Picture rating

Two computer-administered tasks assessed the valuation of high-calorie foods and thin models. Participants rated the palatability of 100 high-calorie food images and the attractiveness of 38 images of thin models.

#### Food liking and craving

The Food Craving Inventory (FCI) (White et al., 2002) assessed craving for high-calorie foods. We also assessed the liking of each food. The original FCI has shown internal consistency (*α* = .93) and 2-week test–retest reliability (*r* = .86) (Martin et al., 2006; White et al., 2002). The adapted food liking scale showed internal consistency and 3-year test–retest reliability in samples of 135 adolescents (*α* = .92; ICC = .86) and 162 adolescents (*α* = .83; ICC = .88); *α* = .91 for food liking and .86 for food craving at baseline, respectively.

#### Additional measures

The supplementary material section provides information on additional risk factors assessed at baseline. We assessed attentional bias for high-calorie foods and thin models with two dot-probe tasks (adopted from Shafran et al., 2007). Pairs of images (20 high-calorie food versus glasses of water pairs; 20 thin-model versus average-weight model pairs) were presented for 500 ms side by side, preceded by a fixation cross for 500 ms. After the images disappeared, a dot probe replaced one image. Participants indicated as quickly as possible whether the probe appeared on the left or right by pressing the response keys. It appeared in the location occupied by a high-calorie food image or thin model 50% of the time. A faster response when the probe replaced high-calorie foods versus glasses of water and thin models versus average weight models provided a behavioral measure of attentional bias. BMI (kg/m^2^) reflects height-adjusted body weight (Pietrobelli et al., 1998). Height was measured to the nearest mm using stadiometers. Weight was assessed to the nearest 0.1 kg using digital scales with participants wearing light indoor clothing without shoes. Immediate reward bias for food was assessed with an adapted hypothetical delay discounting food paradigm (Sellitto et al., 2010); elevated immediate reward bias for food assessed by this paradigm correlated with percent body fat (*r* = .44). Participants were presented with 5 hypothetical snack choices at 6 delays (2 days, 2 weeks, 1 month, 3 months, 6 months, 1 year) plus 10 control trials (choice between 2 different amounts of food, both received ‘now’). Emotionality was assessed with the Emotionality Scale (Buss & Plomin, 1984). Caloric deprivation was measured with a computer program that texted participants 10 times over 10 days at randomly selected times and asked how long it had been since their last intake of caloric foods or beverages. The average time since the last caloric intake from this EMA data was used to estimate habitual caloric deprivation. Birth complications were assessed with a modified version of the Birth and Neonate sections of the Pregnancy History Instrument-Revised (Buka et al., 2000).

### Statistical methods

#### Onset of eating pathology analysis

Logistic regression models, estimated with a logit link tested whether baseline variables predicted the onset of binge eating or compensatory weight control behaviors over 4-year follow-up; odds ratios and 95% confidence intervals are reported. We confirmed the assumption of linearity between continuous predictors and the log odds of the onset of eating disorder behaviors. A post-hoc power analysis showed that with an *N* = 88, assuming a 2-tailed test with an alpha of 0.05, we had adequate power (>0.80) to detect an odds ratio of 2.23 or greater for continuous baseline risk predictors and an odds ratio of 3.89 or greater for the dichotomous baseline risk predictor.

#### MRI acquisition and fMRI data preprocessing

fMRI data acquisition and preprocessing procedures are detailed in the Supplementary Material.

#### fMRI data analysis

To evaluate the relation of blood oxygen level-dependent (BOLD) response to the future onset of eating-disordered behaviors, we conducted a mixed, between-/within-subjects 2 × 2 analyses of variance (e.g., onset of eating-disordered behaviors versus no onset by thin models versus average-weight models). Hunger was included as a covariate of no interest in the tasks involving food stimuli. Analysis of the imaging data focused on three composite regions of interest (ROI) masks using the Wake Forest University Pickatlas toolbox (Maldjian et al., 2003): reward-sensitive brain regions (striatum, medial orbitofrontal cortex [OFC]), inhibitory control regions (ACC, dlPFC, vlPFC, and IFG), and an emotion processing region (bilateral amygdala). Peaks were considered significant at familywise error rate (pFWE) corrected across each ROI mask (see Supplemental Material). Whole brain analyses were also conducted to test for significant activation outside the ROI masks. Results indicated that activity surviving a threshold of *p* ≤ .001 with a cluster *k* ≥ 34 contiguous voxels being statistically significant at FWE-corrected *p* = .05. fMRI effect sizes (*r*) were derived from the *Z*-values (*Z*/√*N*). We confirmed that effects were not driven by influential outliers using regression diagnostics (e.g., residual plots, leverage, Cook’s distance) for the logistic regression models and visualization for the fMRI data. No influential outliers were identified.

## Results

Among the asymptomatic baseline sample, 17 (19%) showed the future onset of binge eating or compensatory weight control behaviors over the 4-year follow-up (5 [6%] binge eating onset and 15 [17%] compensatory behavior onset). Attrition was 7% at the 1-year follow-up, 6% at the 2-year follow-up, 8% at the 3-year follow-up, and 16% at the 4-year follow-up. Most participants (75%) had all four follow-up assessments, 16% had three, 7% had two, and 2% had one follow-up assessment. The number of follow-up assessments was not significantly related (at *p* < 0.05) to demographic characteristics but was related to two baseline risk factors. Participants who did not provide completed follow-up data reported higher baseline caloric deprivation (*d* = 0.74, *p* = 0.016) and lower thin-ideal internalization (*d* = 0.92, *p* = 0.006) than those with complete data. Correlations between the risk factors are shown in Supplementary Material and confirm that these factors were generally only minimally correlated (mean *r* = 0.04, *r* = −0.37 to 0.58). At baseline, 17 participants reported current use of hormonal contraceptives; use at baseline did not predict the future onset of binge eating or compensatory weight control behaviors.

**Figure 1.**
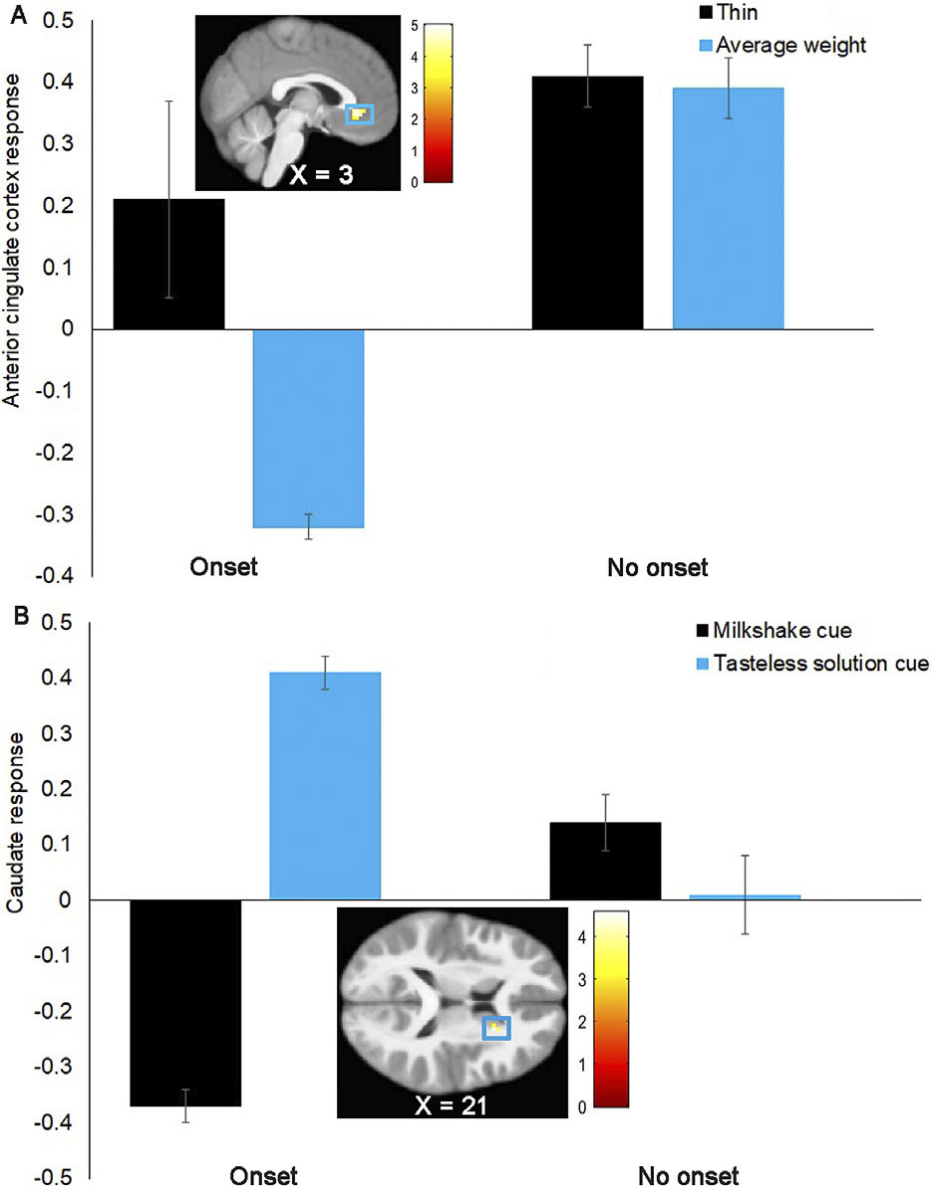
(a) Participants reporting the onset of eating-disordered behaviors (*n* = 16) versus no onset (*n* = 63) showed greater blood-oxygen-level-dependent (BOLD) response in the right anterior cingulate cortex (ACC MNI coordinates: 3, 32, −7, *Z* = 4.80, *k* = 34; *r* = 0.54) in response to the contrast thin models > average-weight models. (b) Participants reporting the onset of eating-disordered behaviors (*n* = 16) versus no onset (*n* = 66) showed less BOLD response in the right caudate (MNI coordinates: 21, 14, 14, *Z* = 4.42, pFWE = 0.008; *r* = 0.49) in response to the contrast milkshake cue > tasteless solution cue. Units on the *y*-axis represent mean parameter estimates of the BOLD signal from the local peak response.

Group differences in neural response to thin versus average-weight models were nonsignificant in the hypothesized ROI. Whole brain analyses showed that participants reporting onset of eating-disordered behaviors versus no onset showed greater right dorsal anterior cingulate cortex response (dACC MNI coordinates: 3, 32, −7, *Z* = 4.82, *k* = 34; *r* = .54; Figure 1A), extending into the left ventromedial prefrontal cortex (vmPFC MNI coordinates: −3, 29, −13, *Z* = 3.63; *r* = 0.41) to the thin model > average-weight model contrast. Because parental-history-positive versus negative youth in the present sample showed differential neural response in some of the fMRI tasks (Stice et al., 2021b), we conducted sensitivity analyses to test whether the predictive effects changed when we covary for parental history of eating pathology. The predictive effects remained the same, suggesting the predictive effects were independent. We also conducted sensitivity analyses to test whether the results remained significant when excluding participants reporting hormonal contraceptive use at baseline *(n* = 17). For these analyses, we extracted the main effect parameter estimates at the individual level (*n* onset = 12; *n* no onset = 54) from the significant peak coordinates from SPM and exported these to Statistical Package for the Social Sciences (SPSS; SPSS Inc) to conduct the analyses. The results remained significant (ACC *r* = 0.46, *p* < 0.001).

Participants reporting the onset of eating-disordered behaviors versus no onset showed less activation in the right caudate (MNI coordinates: 21, 14, 14, *Z* = 4.43, *p*FWE = 0.007, *r* = −0.49; Figure 1B) in response to the milkshake cue > tasteless solution cue contrast. Sensitivity analyses showed that the predictive effect remained the same when including parental history of eating pathology as a covariate. Sensitivity analyses also showed that the predictive effect remained when excluding participants reporting hormonal contraceptive use at baseline (*r* = −0.58, *p* < 0.001). There were no significant group differences in neural response to the milkshake receipt > tasteless solution receipt contrast.

There were no significant differences between groups in response to the food image, food go/no-go, and negative mood induction paradigms.

Five of the 16 baseline risk factors predicted the future onset of binge eating or compensatory weight control behaviors; emotionality (OR = 5.62), *z*-score BMI (OR = 2.91), overvaluation of weight or shape (OR = 5.82), feeling fat (OR = 2.08), and parental history of eating disorder symptoms (OR = 4.42) (Table 2). Benjamini–Hochberg false discovery rate adjusted *p*-values (Benjamini & Hochberg, 1995) were computed for all 16 logistic regression models and all five statistically significant unadjusted *p*-values remained statistically significant after adjustment (all five *p*-values <.036) for multiple testing.

**Table 2.** Results of logistic regression models predicting the onset of future binge eating or compensatory weight control behaviors (*N* = 88)

Baseline risk predictor	Beta	SE	*p*-value	OR	95% CI
Birth complications	0.01	0.03	0.632	1.01	0.96–1.07
Caloric deprivation	0.21	0.21	0.305	1.23	0.82–1.85
Delayed discounting	0.50	1.11	0.650	1.67	0.19–14.62
Dot probe					
Difference thin average models	0.01	0.01	0.616	1.01	0.98–1.04
Difference between unhealthy and healthy foods	−0.01	0.02	0.493	0.99	0.96–1.02
Emotionality	**1.73**	**0.50**	**<**0**.001**	**5.62**	**2.11–15.00**
Food craving	0.20	0.47	0.668	1.22	0.49–3.04
Food liking	−1.28	0.79	0.108	0.28	0.06–1.33
Picture rating					
Thin models	−0.08	0.18	0.639	0.92	0.65–1.30
High calorie foods	−0.18	0.22	0.402	0.83	0.55–1.28
Thin ideal internalization	−0.25	0.41	.776	0.78	0.35–1.72
zBMI	**1.07**	**0.38**	0**.005**	**2.91**	**1.38–6.14**
Overvaluation of weight/shape	**1.76**	**0.42**	**<**0**.001**	**5.82**	**2.54–13.34**
Fear of weight gain	1.07	0.72	0.134	2.92	0.72–11.88
Feeling fat	**0.73**	**0.23**	0**.001**	**2.08**	**1.33–3.26**
Parental history of eating disorder symptoms	**1.49**	**0.59**	0**.011**	**4.42**	**1.40–13.97**

*Note*: SE = standard error, OR = odds ratio, zBMI = *z*-scores body mass index, CI = confidence interval. Results significant at *p* < 0.05 are bolded.

## Discussion

Participants who reported the future onset of binge eating or compensatory weight control behaviors versus no onset showed greater neural response in the dACC, extending into the vmPFC, in response to thin models. Both the dACC and vmPFC are implicated in reward-based decision-making (Chau et al., 2018). Prior research found that participants showed greater vmPFC activation when viewing attractive faces versus less attractive faces (Pegors et al., 2015; Winston et al., 2007). The current findings extend evidence that ACC activation in response to food images and vmPFC activation in response to winning money correlate with binge eating behaviors (Bodell et al., 2018; Schienle et al., 2009). Also consistent with hypotheses, overvaluation of weight/shape and feeling fat predicted the future onset of eating-disordered behaviors, but not fear of weight gain. Results converge with previous evidence that valuation of the thin ideal and overvaluation of weight/shape predicted the future onset of eating disorders (Stice et al., 2021a). Results are also consistent with the finding that parental-history-positive versus negative youth in the present sample reported greater overvaluation of weight/shape and feeling fat (Stice et al., 2021b). Results suggest that the valuation of the thin ideal increases the risk for the onset of eating-disordered behaviors. However, self-reported thin-ideal internalization, attractiveness ratings of thin models, and attentional bias for thin models did not predict the onset of eating-disordered behaviors.

Participants who reported the future onset of binge eating or compensatory weight control behaviors versus no onset showed lower caudate activation in response to anticipated high-calorie tastes. This finding is seemingly inconsistent with the finding that parental-history-positive versus negative youth in the present sample showed elevated putamen response to anticipated high-calorie tastes (Stice et al, 2021b). To better interpret this finding, we conducted exploratory analyses to test whether the caudate peak correlated with any of the 5 self-reported risk factors (i.e., emotionality, overvaluation of weight or shape, feeling fat, and parental history of eating disorder symptoms). There was a significant negative correlation between caudate activation in response to anticipated taste and feeling fat (*r* = −0.27, *p* = 0.01). This suggests that the reduced caudate activation in response to anticipated tastes of high-calorie foods in females reporting the onset of eating-disordered behavior can, in part, be explained by feeling fat. This result extends prior research showing lower striatal activation to tastes of high-calorie foods in women with or recovered from AN compared with healthy controls (Wagner et al. 2008). Elevated zBMI also predicted the future onset of eating-disordered behaviors, consistent with the hypothesis that overeating increases the risk for the emergence of binge eating and compensatory behaviors. This result converges with evidence that elevated BMI predicts the future onset of PD (Yamamiya & Stice, 2024). Elevated zBMI may have contributed to the predictive effects of overvaluation of weight and shape and feeling fat.

We did not find evidence that lower activation of inhibitory control regions in response to tastes and anticipated tastes of chocolate milkshake or the food go/no-go paradigm predicted the future onset of binge eating or compensatory weight control behaviors. The fact that individuals with BN or BED show consistent deficits in inhibitory control (e.g., Hege et al., 2015; Skunde et al., 2016), but that youth with parental history of eating pathology did not show evidence of inhibitory control deficits and that there was no evidence that deficits in inhibitory control predicted the future onset of binge eating or compensatory behaviors in the present study suggests that engaging in impulsive behaviors on a habitual basis (recurrent binge eating) might reduce inhibitory control.

There were no group differences in neural response to the negative mood induction. However, self-reported emotionality predicted the future onset of eating-disordered behaviors. This pattern of findings suggests that the self-report emotionality measure may be more sensitive than the negative mood induction task. The predictive effect of self-reported emotionality on future eating-disordered behaviors dovetails with evidence that negative affect predicts the future onset of eating disorders (Dakanalis et al., 2017; Killen et al., 1996; Stice et al., 2017). Further, parental-history-positive versus negative youth in the present sample reported higher emotionality than parental-history-negative youth at baseline (Stice et al., 2021b). Overall, results suggest that higher emotionality may increase the risk for future onset of eating-disordered behaviors.

Results did not provide evidence that elevated caloric deprivation, based on EMA, predicted the future onset of eating disorder behaviors. This null finding is noteworthy given evidence that parental history positive versus negative youth reported elevated caloric deprivation at baseline (Stice et al., 2021b) and that self-reported dietary restraint predicted the future onset of eating disorders (e.g., Stice et al., 2021a). It is possible that self-reported hours since the last caloric intake shows limited test–retest reliability, which might have made it difficult to observe predictive effects.

Parental history of binge eating or compensatory weight control behaviors predicted the future onset of binge eating and compensatory behaviors in adolescents over follow-up. This finding extends evidence that children of parents with a lifetime history of an eating disorder exhibit elevated eating disorders (Bould et al., 2015; Lydecker & Grilo, 2016), providing further evidence that parental history-positive youth are at elevated risk for eating pathology.

There was no evidence that birth complications increase the risk for the future emergence of binge eating and compensatory behaviors. This result contrasts with a previous study that found a correlation between birth defects and a lifetime history of eating disorders (Cnattingius et al.,1999). Birth complications were also not correlated with parental history of eating pathology (Stice et al., 2021b). Thus, results suggest that birth complications do not increase the risk for eating-disordered behaviors.

To our knowledge, this is the first fMRI study to test whether individual differences in neural responsivity predict the future onset of eating-disordered behaviors. The 4-year follow-up is another positive design feature. However, there are study limitations. First, the moderate sample size limited the power to detect small effects. It would be useful to investigate the examined risk factors in a larger prospective study. Second, we were not able to predict the future onset of binge eating and compensatory weight control behaviors in separate models. Third, we required that participants not meet the criteria for an Axis I psychiatric disorder at baseline to rule out potential confounds. However, this makes this sample somewhat unrepresentative of youth in general and youth at risk for eating disorders in particular, which should be considered when interpreting the findings. It might be useful for future larger risk factor studies to include such individuals and test whether a history of psychiatric disorders moderates the relationship between risk factors and eating pathology onset. Fourth, we did not assess parental binge eating and compensatory weight control behaviors over follow-up, which may have changed over time. Likewise, we did not assess hormonal contraceptive use over follow-up. Finally, although we focused on a broad range of risk factors, we were not able to measure other potential risk factors, such as social media use, food insecurity, and individual differences in gut microbiota.

In sum, the evidence that elevated valuation of the thin ideal and lower anticipatory food reward increase the risk for the future onset of binge eating or compensatory behaviors are novel contributions to the literature and suggest that individual differences in neural responsivity represent potential biological risk factors for eating disorder behaviors. The evidence that overvaluation of weight/shape, feeling fat, elevated body mass, emotionality, and parental history of eating pathology predicted the future onset of binge eating or compensatory behaviors extends prior findings. Results suggest that prevention programs should focus on reducing the valuation of the thin ideal, appearance overvaluation, emotionality, and overeating, which dovetails with evidence that prevention programs that target the former and latter factors reduce future eating disorder onset (Stice et al., 2021c). Future research should seek to replicate these findings in larger samples and explore other potential eating disorder risk factors.

## Supporting information

Stice et al. supplementary material Stice et al. supplementary material

## Supplementary material

The supplementary material for this article can be found at http://doi.org/10.1017/S0033291724003337.
