# Supplementary material for: Neural risk factors that predict the future onset of binge eating or compensatory weight control behaviors: A prospective 4-year fMRI study
Source: Psychol Med. 2025 Feb 17;55:e48. doi: 10.1017/S0033291724003337 (PMC12055025; doi:10.1017/S0033291724003337)
Supplement: Stice et al. supplementary material [file S0033291724003337sup001.docx]

**Neural Risk Factors that Predict Future Onset of Binge Eating and Compensatory Weight Control Behaviors: A Prospective 4-Year fMRI Study**

Eric Stice

Stanford University

Sonja Yokum

Jeff Gau

Oregon Research Institute

Heather Shaw

Stanford University

**Supplementary Methods**

**Non-fMRI Measures**

**Dot probe.** Participants completed dot-probe tasks that assessed attentional bias for high-calorie foods and thin models. In these paradigms (adopted from Shafran et al., 2007), pairs of images (20 high-calorie food vs glasses of water pairs; 20 thin-model vs average-weight model pairs) was presented for 500ms side by side, preceded by a fixation cross for 500ms. Right after the images disappeared, a dot probe replaced one image. Participants indicated as quickly as possible whether the probe appeared on the left or right by pressing response keys. It appeared in the location occupied by a high-calorie food image or thin model 50% of the time. A faster response when the probe replaced high-calorie foods versus glasses of water and thin models versus average weight models provided a behavioral measure of attentional bias. Each of the 20 picture pairs were presented 6 times (120 trials), with each picture presented 3 times on each side. Response times less than 150ms or more than 1,000ms were considered as anticipatory and delayed, respectively, and eliminated (MacLeod et al., 1986). Dot-probe tasks with food stimuli have shown internal reliability (α = 0.79) and test-retest reliability (r = .84) for reaction time bias scores (van Ens et al., 2019). In addition, dot-probe tasks with food and weight stimuli showed greater attentional biases in women with eating disorders versus controls (Shafran et al., 2007).

**Body mass.** BMI (kg/m^2^) reflects height adjusted body weight (Pietrobelli et al., 1998). Height was measured to the nearest mm using stadiometers. Weight was assessed to the nearest 0.1 kg using digital scales with participants wearing light indoor clothing without shoes. Each was measured twice and averaged. BMI has shown convergent validity (*r*=.80–.90) with body fat measures (Pietrobelli et al., 1998). We analyzed age- and sex-based BMI z-scores because participants were still growing.

**Delay discounting.** We adapted the delay discounting food paradigm from Sellitto et al. (2010). Participants were presented with 5 hypothetical snack choices at 6 delays (2 days, 2 weeks, 1 month, 3 months, 6 months, 1 year) plus 10 control trials (choice between 2 different amounts of food, both received “now”). During the task, 2 snack amounts appeared on the screen with 2 different time points (“now” and after a delay). In each block, the delayed amount was 40 units (e.g., 40 chocolate bars). Amount of the immediate reward was adjusted using a staircase procedure that converged on the amount of immediate reward being equal in value to delayed reward. Participants completed each delay for a salty and a sweet snack that they selected out of 10 options. The rate at which the subjective value of the food reward decays with delay (k) provided a behavioral measure of delay discounting. The food delay discounting paradigm has shown convergent validity with alternative measures (Hendrickson et al., 2015).

**Emotionality.** Participants completed the Emotionality Scale (Buss & Plomin, 1984), which assesses agreement with 8 items reflecting emotionality (e.g., “I frequently get upset”) over the last month, the same assessment window used for the measures below unless otherwise indicated. It has shown internal consistency (α=.82), 1-month test-retest reliability (*r*=.80), convergent validity, and predictive validity (Buss & Plomin, 1984; Killen et al., 1996) (α=.82 at baseline).

**Caloric deprivation.** A computer program texted participants 10 times over 10 days at randomly selected times asking how long it had been since their last intake of caloric foods or beverages. Average time since last caloric intake from this EMA data was used to estimate habitual caloric deprivation. As noted, average number of hours since last caloric intake correlated (mean *r*=.53) with fMRI-assessed responsivity of brain reward regions to tastes and anticipated tastes of chocolate milkshake and high-calorie food images (Stice et al., 2013). Adolescents with versus without parental history of eating pathology reported significantly more hours since last caloric intake at baseline (Stice et al., 2021).

**Birth complications.** A modified version of the Birth and Neonate sections of the Pregnancy History Instrument-Revised (Buka et al., 2000) assessed birth complications. This interview was adapted into an 18-item parent-report questionnaire to assess birth complications that correlated with a lifetime history of AN (Cnattingius et al., 1999) and related complications. Data indicate that mothers can accurately recall childbirth information over 22 years later (Buka et al., 2004).

**fMRI data acquisition**

Data were acquired using a Siemens Skyra 3 Tesla MRI scanner. A 32-channel head coil acquired data from the entire brain. Functional scans used a T2* weighted echo-planar plus sequence (72 slices, TE = 25 ms, TR = 2000 ms, flip angle = 90°, matrix size = 100 x 100, voxel size = 2 mm^3^, axial slices = 72, FOV = 200). Structural scans were collected using a high-resolution anatomical T1-weighted MP-RAGE scan (TE = 3.43 ms, TR = 2500 ms, 256 x 256 matrix, voxel size = 1 mm^3^, sagittal slices = 176, FOV = 256).

**fMRI data preprocessing**

DICOM images were converted to NIfTI format via MRIConvert ([http://lcni.uoregon.edu/~ jolinda/MRIConvert/](http://lcni.uoregon.edu/~%20jolinda/MRIConvert/)) and non-brain tissue was removed using Brain Extraction Tool in FSL (FMRIB Analysis Group, Oxford, UK). Data were preprocessed and analyzed using statistical parametric mapping (SPM12; Wellcome Department of Imaging Neuroscience) in MATLAB. Anatomical data were segmented and normalized to Montreal Neurological Institute space with the use of the DARTEL toolbox, resulting in a sample-specific template and individual-level deformation fields for application to the normalization step during functional data preprocessing. Functional data were adjusted for variation in magnetic field distortion using field maps, realigned to the mean, coregistered with the anatomical, normalized to Montreal Neurological Institute (MNI) space with the use of DARTEL and smoothed to 6 mm Gaussian full-width-at-half-maximum (FWHM).

Functional data were assessed to detect spikes in global mean response and motion outliers using the Artifact Detection Toolbox (ART; Gabrieli Laboratory, McGovern Institute for Brain Research, Cambridge MA). Head motion greater than 3 mm or degrees in any direction was our *a priori* exclusion criteria. Motion parameters less than 3 mm were included as regressors in the design matrix at individual-level analysis. One participant did not complete the scan (she felt claustrophobic) and another participant showed excessive movement during all paradigms, resulting in unusable data. For each paradigm, we excluded scan data of the following number of participants: food image n = 5 (excessive movement); food receipt n = 1 (excessive movement); food go/no-go n = 11 (n = 3 excessive movement, n = 8 paradigm dysfunction [missing output files containing information on onset, duration, and behavioral responses]); negative mood induction n = 4 (paradigm dysfunction [paradigm froze or no sound]); model image n = 2 (excessive movement). The following number of participants were included in the main analyses: model image n = 79 (16 onset and 63 no onset), food receipt n = 82 (16 onset and 66 no onset), food image n = 77 (13 onset and 64 no onset); food go/no-go n = 71 (14 onset and 57 no onset); negative mood induction n = 78 (15 onset and 63 no onset). Of the twelve participants showing lower-than-expected body weight at follow-up, 4 were excluded from the exploratory fMRI analyses due to excessive motion during the paradigms.

**fMRI data analysis**

First-level analyses included estimation of event-related condition effects using a general linear model and convolving the canonical hemodynamic response function with stimulus events. Separate regressors were entered for conditions of interest. T-maps were constructed for comparisons of activation within participants for each examined contrast.

Analysis of the imaging data focused on three composite regions of interest (ROI) masks. For the model image paradigm, food receipt paradigm, and the food image paradigm, we created a mask that included 8 (bilateral) reward-sensitive regions: the caudate, nucleus accumbens, putamen, and medial orbitofrontal cortex (OFC). For the food go/no-go paradigm, we created a mask including 8 (bilateral) prefrontal regions implicated in inhibitory control: ACC, dlPFC, vlPFC, and IFG. For the negative mood induction paradigm, we created an ROI including bilateral amygdala (emotion processing region). Peaks were considered significant at familywise error rate (pFWE) corrected across each ROI mask.

**Supplementary Results**

**Table S1**

*Correlations Between Baseline Risk Factors*

|  | | 1. | 2. | 3. | 4. | 5. | 6. | 7. | 8. |
| --- | --- | --- | --- | --- | --- | --- | --- | --- | --- |
| 1. | Birth complications | 1 |  |  |  |  |  |  |  |
| 2. | Caloric deprivation | .03  (.770) | 1 |  |  |  |  |  |  |
| 3. | Delayed discounting | .08  (.487) | -.07  (.563) | 1 |  |  |  |  |  |
| 4. | Dot probe – difference thin avg models | .17  (.118) | -.04  (.752) | .06  (.579) | 1 |  |  |  |  |
| 5. | Dot probe – difference unhealthy and healthy foods | **.22**  **(.044)** | .14  (.229) | -.16  (.151) | .07  (.542) | 1 |  |  |  |
| 6. | Emotionality | .01  (.958) | .14  (.201) | .12  (.289) | -.02  (.882) | -.06  (.605) | 1 |  |  |
| 7. | Food craving | .00  (.986) | -.13  (.236) | **-.28**  **(.008)** | -.04  (.726) | -.03  (.815) | .13  (.232) | 1 |  |
| 8. | Food liking | -.16  (.139) | -.19  (.082) | **-.24**  **(.025)** | -.05  (.637) | -.05  (.644) | -.03  (.817) | **.44**  **(<.001)** | 1 |
| 9. | Attractiveness rating of thin models | .05  (.668) | .06  (.616) | -.14  (.194) | -.03  (.766) | -.10  (.370) | .03  (.820) | .12  (.281) | .18  (.090) |
| 10. | Palatability rating of high calorie foods | -.09  (.397) | -.15  (.170) | **-.32**  **(.003)** | -.19  (.076) | -.16  (.149) | .09  (.418) | **.49**  **(<.001)** | **.58**  **(<.001)** |
| 11. | Thin ideal internalization | .10  (.357) | -.13  (.258) | .09  (.422) | -.06  (.588) | -.18  (.106) | .14  (.203) | .13  (.235) | .06  (.592) |
| 12. | zBMI | .17  (.122) | .10  (.353) | -.20  (.067) | .15  (.176) | .13  (.253) | .08  (.448) | .07  (.513) | -.10  (.369) |
| 13. | Overvaluation of weight/shape | .00  (.971) | .21  (.053) | .05  (.634) | -.05  (.614) | -.09  (.409) | **.58**  **(<.001)** | .14  (.200) | -.09  (.405) |
| 14. | Fear of weight gain | -.02  (.890) | -.07  (.557) | -.07  (.527) | .09  (.401) | **-.37**  **(<.001)** | .15  (.172) | .17  (.110) | .18  (.100) |
| 15. | Feeling fat | -.04  (.715) | **.23**  **(.034)** | .11  (.328) | -.12  (.265) | .04  (.720) | **.48**  **(<.001)** | -.05  (.626) | -.19  (.079) |
| 16. | Parental history of eating disorder symptoms | .07  (.519) | **.29**  **(.008)** | .09  (.401) | -.06  (.585) | -.10  (.348) | **.41**  **(<.001)** | .01  (.904) | **-.24**  **(.026)** |
| Notes. Top number in each cell is the bivariate correlation and second parenthetical number the associated *p*-value. | | | | | | | | | |

**Table S1 (continued)**

|  | | 9. | 10. | 11. | 12. | 13. | 14. | 15. | 16. |
| --- | --- | --- | --- | --- | --- | --- | --- | --- | --- |
| 1. | Birth complications |  |  |  |  |  |  |  |  |
| 2. | Caloric deprivation |  |  |  |  |  |  |  |  |
| 3. | Delayed discounting |  |  |  |  |  |  |  |  |
| 4. | Dot probe – difference thin avg models |  |  |  |  |  |  |  |  |
| 5. | Dot probe – difference unhealthy and healthy foods |  |  |  |  |  |  |  |  |
| 6. | Emotionality |  |  |  |  |  |  |  |  |
| 7. | Food craving |  |  |  |  |  |  |  |  |
| 8. | Food liking |  |  |  |  |  |  |  |  |
| 9. | Attractiveness rating of thin models | 1 |  |  |  |  |  |  |  |
| 10. | Palatability rating of high calorie foods | **.32**  **(.002)** | 1 |  |  |  |  |  |  |
| 11. | Thin ideal internalization | .18  (.101) | -.04  (.690) | 1 |  |  |  |  |  |
| 12. | zBMI | -.07  (.527) | -.04  (.714) | -.01  (.909) | 1 |  |  |  |  |
| 13. | Overvaluation of weight/shape | **.22**  **(.037)** | -.01  (.924) | **.21**  **(.050)** | **.39**  **(<.001)** | 1 |  |  |  |
|  |  |  |  |  |  |  |  |  |  |
| 14. | Fear of weight gain | **.27**  **(.012)** | .17  (.116) | .05  (.638) | -.01  (.962) | **.29**  **(.006)** | 1 |  |  |
| 15. | Feeling fat | .03  (.810) | -.12  (.282) | .06  (.560) | **.27**  **(.013)** | **.51**  **(<.001)** | -.02  (.880) | 1 |  |
| 16. | Parental history of eating disorder symptoms | -.10  (.349) | -.15  (.163) | .02  (.844) | .08  (.470) | **.30**  **(.004)** | -.08  (.455) | **.34**  **(.001)** | 1 |
| Notes. Top number in each cell is the bivariate correlation and second parenthetical number the associated *p*-value. | | | | | | | | | |

**Supplemental References**

Buka, S., Goldstein, J., Seidman, L., & Tsuang, M. (2000). Maternal recall of pregnancy history: accuracy and bias in schizophrenia research. *Schizophrenia Bulletin, 26*, 335-350. https://doi.org/10.1093/oxfordjournals.schbul.a033457

Buka, S., Goldstein, J., Spartos, E., & Tsuang, M. (2004). The retrospective measurement of prenatal and perinatal events: accuracy of maternal recall. *Schizophrenia Research, 71*, 417-426. https://doi.org/10.1016/j.schres.2004.04.004

Buss, A., & Plomin, R. (1984). *Temperament: Early developing personality traits*. L. Erlbaum Associates.

Cnattingius, S., Hultman, C., Dahl, M., & Sparén, P. (1999). Very preterm birth, birth trauma, and the risk of anorexia nervosa among girls. *Archives of General Psychiatry, 56*, 634-638. https://doi.org/10.1001/archpsyc.56.7.634

Hendrickson, K. L., Rasmussen, E. B., & Lawyer, S. R. (2015). Measurement and validation of measures for impulsive food choice across obese and healthy-weight individuals. *Appetite*, *90*, 254–263.

Killen, J., Taylor, C., Hayward, C., Haydel, K., Wilson, D., Hammer, L., et al., (1996). Weight concerns influence the development of eating disorders: a 4-year prospective study. *Journal of Consulting and Clinical Psychology, 64*, 936-940. <https://doi.org/10.1037//0022-006x.64.5.936>

MacLeod, C., Mathews, A., & Tata, P. (1986). Attentional bias in emotional disorders. *Journal of Abnormal Psychology, 95*, 15-20. https://doi.org/10.1037//0021-843x.95.1.15

Pietrobelli, A., Faith, M., Allison, D., Gallagher, D., Chiumello, G., & Heymsfield, S. (1998). Body mass index as a measure of adiposity among children and adolescents: a validation study. *Journal of Pediatrics, 132*, 204-210. https://doi.org/10.1016/s0022-3476(98)70433-0

Sellitto, M., Ciaramelli, E., & di Pellegrino, G. (2010). Myopic discounting of future rewards after medial orbitofrontal damage in humans. *Journal of Neuroscience, 30*, 16429-16436. https://doi.org/10.1523/jneurosci.2516-10.2010

Shafran, R., Lee, M., Cooper, Z., Palmer, R. L., & Fairburn, C. (2007). Attentional bias in eating disorders. *International Journal of Eating Disorders, 40*, 369-380. <https://doi.org/10.1002/eat.20375>

Stice, E., Burger, K., & Yokum, S. (2013). Caloric deprivation increases responsivity of attention and reward regions to intake, anticipated intake, and images of palatable foods. *NeuroImage, 67,* 322-330.

Stice, E., Yokum, S., Rohde, P., Cloud, K., & Desjardins, C. D. (2021). Comparing healthy adolescent females with and without parental history of eating pathology on neural responsivity to food and thin models and other potential risk factors. *Journal of Abnormal Psychology*, *130*(6), 608–619.

van Ens, W., Schmidt, U., Campbell, I. C., Roefs, A., & Werthmann, J. (2019). Test-retest reliability of attention bias for food: Robust eye-tracking and reaction time indices. *Appetite, 136,* 86–92.
